# Effect of COVID-19 Pandemic-Related Delays in Chemoembolization on the Survival of Patients with Hepatocellular Carcinoma

**DOI:** 10.1155/2023/8114732

**Published:** 2023-04-14

**Authors:** Kittipitch Bannangkoon, Keerati Hongsakul, Teeravut Tubtawee, Phurich Janjindamai

**Affiliations:** Department of Radiology, Faculty of Medicine, Prince of Songkla University, Hat Yai, Songkhla 90110, Thailand

## Abstract

**Background and Aims:**

COVID-19 has led to potential delays in liver cancer treatment, which may have undesirable effects on the prognosis of patients. We aimed to quantify the COVID-19 pandemic impact on the survival of patients with hepatocellular carcinoma (HCC) who underwent transarterial chemoembolization (TACE).

**Methods:**

A retrospective study was conducted in patients with HCC who underwent TACE at a tertiary care center during the prelockdown (March to July 2019) and lockdown (March to July 2020) periods. Demographic data, tumor characteristics, functional status, and vital status were collected from the hospital medical records. The endpoints were TACE interval, treatment response, and survival after TACE. Cox proportional hazards regression determined the significant preoperative factors influencing survival.

**Results:**

Compared to prelockdown, a significant delay occurred during the lockdown in repeated TACE treatments (76.7 vs. 63.5 days, *P*=0.007). The trend suggested a significant decrease in patients with HCC in the repeated TACE group (−33.3%). After screening, 145 patients were included (prelockdown (*n* = 87), lockdown (*n* = 58)). There was no significant difference in the 1-month objective response rate between the prelockdown and lockdown groups (65.5% vs. 64.4%, *P*=1.00). During follow-up, 56 (64.4%) and 34 (58.6%) deaths occurred in the prelockdown and lockdown groups, respectively (*P*=0.600). Multivariate analysis revealed no association between the lockdown group and decreased survival (HR 0.88, 95% CI 0.57–1.35, *P*=0.555).

**Conclusions:**

The impact of the COVID-19 pandemic on liver cancer care resulted in significant decreases and delays in repeated TACE treatments in 2020 compared to 2019. However, treatment delays did not seem to significantly impact survival.

## 1. Introduction

Coronavirus disease 2019 (COVID-19) was first reported in Wuhan, China, in late December 2019 and rapidly evolved into a global pandemic. As of April 17, 2022, the cumulative number of cases reported globally was over 500 million, and the cumulative number of deaths was over 6 million [1]. Outside China, Thailand was the first country to report a case of COVID-19 [2]. On January 13, 2020, Thailand reported that a Wuhan resident who traveled to Bangkok had tested positive for COVID-19 [3]. The country entered a lockdown (state of emergency) on March 26, 2020, and announced a nationwide curfew on April 3, 2020, to protect Thai citizens from COVID-19 [3]. Although the number of new cases showed a downward trend, the COVID-19 emergency decree for Thailand was extended to July 31, 2020, to limit the spread of this virus [2, 3]. The rapid spread of COVID-19 impacted the ability of healthcare systems to deliver high-quality and accessible services including cancer care.

Oncology services for patients with cancer were reorganized, and courses of action were considered to balance delays in diagnosis or treatment against the risk of COVID-19. However, routine cancer care was postponed, which resulted in unintended consequences. This may have impacted long-term prognosis and survival outcomes in patients who missed cancer screenings and surgical intervention during this period. Moreover, the World Health Organization reported that in May 2020, 42% of 155 countries had partially or completely suspended cancer treatment and confirmed that the impact was global [4].

An association between increased mortality and delayed treatment has been demonstrated in various types of cancers. A systematic review stated that an association was found between a delay of four weeks in cancer treatment and increased mortality in cancers of the bladder, breast, colon, rectum, lung, cervix, and head and neck [5]. In hepatocellular carcinoma (HCC), the association between survival and treatment delays is a topic that continues to be unsettled. Three studies in Taiwan, Canada, and the United States showed that delays in the treatment of locoregional cancers were associated with survival and poor responses from treatment [6–8]. In contrast, two studies found that patients with treatment delays had better crude survival than patients without delays. Furthermore, survival was not associated with delays after adjustments were made in other prognostic factors [9, 10]. The American Association for the Study of Liver Diseases (AASLD) recommends going ahead with HCC treatments when able rather than delaying the treatments since the benefits likely outweigh the risk of COVID-19 exposure [11]. Therefore, appraising the ramifications of a delay in therapeutics on prognosis and outcomes is necessary.

Our study aimed to evaluate the prevalence of therapeutic delays and characterize the impact of the COVID-19 pandemic on the survival of patients with inoperable HCC who underwent chemoembolization.

## 2. Methods

### 2.1. Ethics Statement

This study complied with the standards of the Declaration of Helsinki and current ethical guidelines and was approved by the institutional ethics committee (REC No. 64-456-7-1). The requirement of obtaining informed consent for this study was waived by the institutional review board, and all data were analyzed anonymously.

### 2.2. Patient Population

This retrospective cohort study was conducted in consecutive patients diagnosed with HCC who underwent transarterial chemoembolization (TACE) between March 1 and July 30, 2019, and during the same period in 2020 at a university-affiliated tertiary care referral center in southern Thailand. All eligible patients who underwent TACE from March 1, 2020, to July 30, 2020, were included in the lockdown group (after COVID-19). These patients were compared with similar patients who underwent TACE in the previous year in the prelockdown group (before COVID-19). The diagnosis of HCC was established using the AASLD criteria based on characteristic imaging features or histopathological confirmation [12]. Tumor staging was achieved using the Barcelona Clinic Liver Cancer (BCLC) staging system [13]. In our study, the inclusion criteria were as follows: adults aged ≥18 years with HCC, patients diagnosed with HCC classified as BCLC stages A and B, patients diagnosed with HCC classified as BCLC stage C with limited portal vein tumor thrombosis in the second-order branches or those more distal, patients with Child–Pugh classes A or B, and patients treated at TACE sessions during the study periods. The exclusion criteria were as follows: patients with liver masses without characteristic imaging or histology, patients who received cotreatment with systemic therapies during the TACE session, patients with severe arterioportal shunt, patients with a history of spontaneous tumor rupture, and patients with incomplete follow-up data. Vital status and date of death were assessed through the National Health Service database up to March 31, 2021, and March 31, 2022, for the prelockdown and lockdown groups, respectively.

### 2.3. Data Collection

We obtained patient characteristics, clinical history, laboratory data, and imaging results at HCC presentation and treatment procedures from the electronic medical records. Variables of interest included age, sex, liver disease etiology, Child–Pugh class, BCLC stage, tumor burden, vascular invasion, number of TACE sessions, TACE interval, and length of hospital stay. Laboratory data at the pre-TACE session included alanine transaminase (ALT), aspartate transaminase (AST), bilirubin, albumin, platelet count, and alpha-fetoprotein (AFP).

### 2.4. TACE Protocols

In all patients, the TACE procedure was performed under local anesthesia through the transfemoral route by two interventional radiologists, each with more than eight years of experience in HCC therapy. First, visceral angiography of the celiac and superior mesenteric arteries was routinely performed to assess vascular anatomy, tumor vascularity, and portal vein patency using a 5-F catheter. Second, we performed selective catheterization to the tumor-feeding hepatic arteries or in extrahepatic collaterals as distal as possible in each tumor lesion using a microcatheter of either a 1.7-Fr microcatheter (Terumo, Tokyo, Japan) or 1.98-Fr tip microcatheter (Asahi Intecc, Nagoya, Japan) over a 0.016-inch or a 0.018-inch guidewire. Subsequently, a mixture of 10–20 mg of mitomycin C (Atlanta Medicare, Bangkok, Thailand) and 4–16 mL of iodized oil (Lipiodol Ultra-Fluide; Laboratoire Andre Guerbet, Aulnay-sous-Bois, France) was injected into the tumor for chemoembolization. The amount of anticancer-in-oil emulsion was determined by tumor size, number of nodules, and arterial supply to the tumor. Subsequently, the feeding artery was embolized using gelatin sponge particles. Finally, we completed the procedure when the tumor-feeding branch was completely obstructed, and tumor staining from digital subtraction angiography (Allura Clarity FD20, Philips Healthcare, the Netherlands) completely disappeared.

### 2.5. Follow-Up and Repeat TACE

Enhanced computed tomography or magnetic resonance imaging was performed four weeks after TACE by experienced radiologists to assess the radiological tumor response according to the modified Response Evaluation Criteria in Solid Tumors (mRECIST) criteria [14]. Detailed clinical examinations, blood chemistry tests, and chest radiography were also performed during the follow-up visits. If no definite evidence of a residual or recurrent tumor was present, the imaging study was performed subsequently at 3-month intervals. The decision to repeat the TACE procedure was made by a multidisciplinary team of experts, including a radiologist, interventional radiologists, hepatologists, oncologists, and surgeons, based on the tumor response according to the mRECIST criteria, BCLC staging of the disease, and patient tolerance. Generally, repeated TACE was performed on demand at 6- to 8-week intervals.

The first TACE interval was defined as the duration from the date of HCC diagnosis to the date of the initial TACE procedure. In patients undergoing repeated TACE, the TACE interval was classified as the duration from the date of the latest TACE or latest recurrent imaging (before study periods) to the date of the TACE procedure during the study periods (March to July 2019 and March to July 2020). First TACE delays were defined as 90 days from diagnosis to initial treatment. Repeated TACE delays were also defined as 90 days from the latest TACE or latest recurrent imaging to the date of the TACE procedure during the study periods. The 90-day cutoff value was selected as a clinically relevant time point based on prior literature [6, 15]. We also gathered information on the reasons for treatment delays and alternative therapeutic decisions.

### 2.6. Statistical Analyses

Statistical analyses were performed using *R* software version 4.1.0. Continuous data are expressed as mean or median with a measure of dispersion (standard deviation and range), and comparisons were achieved using Student's *t*-test or the Mann–Whitney *U* test. Fisher's exact test was used to compare categorical data. Overall survival (OS) was calculated from the date of TACE to the date of patient death or the last follow-up date. Patient status at the end of each study period (March 31, 2021, and March 31, 2022) was defined as alive or dead. Baseline clinical characteristics, tumor manifestations, and lockdown grouping affecting survival were initially assessed via univariate analysis. Subsequently, all prognostic factors with a *P* value ≤0.2 from the univariate analysis were entered into the multivariate Cox proportional hazards regression analysis. The model was refined by sequentially removing nonsignificant variables. *P* values <0.05 were considered statistically significant.

## 3. Results

### 3.1. Patient Inclusion/Exclusion Criteria

A total of 262 patients with HCC treated with TACE between March and July 2019 and during the same period in 2020 at Songklanagarind Hospital in southern Thailand were included in this study. Twenty-two patients were excluded for the following reasons: incomplete follow-up data (*n* = 8); absence of HCC mass (*n* = 5); history of spontaneous tumor rupture (*n* = 5); cotreatment with systemic therapies during TACE session (*n* = 3); and severe arterioportal shunt (*n* = 1). Subsequently, 240 patients were enrolled. Among them, 95 (39.6%) and 145 (60.4%) patients underwent the first TACE and repeated TACE procedures, respectively.

### 3.2. Impact of the Pandemic on HCC Patients Who Underwent TACE

**Figure 1:**
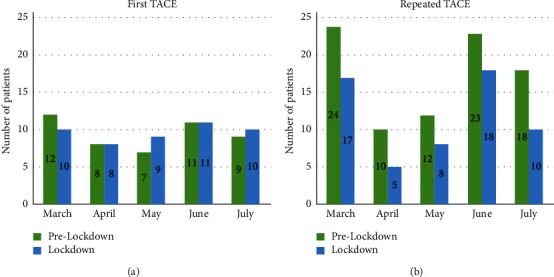
Numbers of patients with hepatocellular carcinoma during the prelockdown (March to July 2019) and lockdown (March to July 2020) periods who underwent first transarterial chemoembolization (TACE) (a) and repeated TACE (b).

Initially, a similar number of patients who underwent the first TACE were examined during the lockdown (*n* = 48) and prelockdown (*n* = 47) periods (Figure 1(a)). However, there was a significant decrease in the number of patients who underwent repeated TACE during the lockdown period (*n* = 58) compared to the prelockdown period (*n* = 87), which represented a 33.3% reduction. Repeated TACE services dropped every month from March to July 2020: March (−29%), April (−50%), May (−33%), June (−22%), and July (−44%) (Figure 1(b)). The mean first TACE interval was not significantly different between the two groups (51.2 vs. 46.5 days, *P*=0.826) (Table 1). However, the mean TACE interval in patients who underwent repeated TACE in the lockdown group was significantly longer than in the prelockdown group (76.7 vs. 63.5 days, *P*=0.007). First TACE delay and repeated delay >90 days in the lockdown group tended to be higher than the prelockdown group, but not significantly different (14.6% vs. 4.3%, *P*=0.159, and 22.4% vs. 11.5%, *P*=0.126).

### 3.3. Cause of Delayed TACE (>90 Days) in the Lockdown Group

A total of 20 cases (13 in the repeated TACE group and 7 in the first TACE group) experienced delays in the TACE procedure of more than 90 days. The causes of TACE delay were reported as follows: 12 cases were delayed due to healthcare providers postponing the TACE procedure, 4 cases were delayed due to poor liver function, 3 cases were delayed due to delayed imaging or diagnosis, and 1 case was delayed due to patient noncompliance (transportation limitations).

### 3.4. Baseline Characteristics of Patients in the Repeated TACE Group

The basic characteristics of the recruited patients who underwent repeated TACE are summarized in Table 2. This study enrolled 145 HCC patients (male: 75%) who underwent repeated TACE with a mean ± SD age of 64.0 ± 9.7 years. The major etiologies of HCC were hepatitis B virus (HBV) (55%), hepatitis C virus (24%), and alcohol (6%). The Child–Pugh scores of liver cirrhosis were classified as A (78%) and B (22%). The median size of the main tumors was 3.0 cm in diameter (range: 1.1–16.0 cm in diameter). About half of the patients (55%) had tumors with fewer than three nodules. According to the BCLC system, the percentages of cases classified as BCLC stage A, B, and C were 14%, 73%, and 13%, respectively. The median (IQR) values for baseline serum ALT and AST were 32.0 (23.0–43.0) U/L and 49.0 (34.0–76.0) U/L, respectively. The mean ± SD serum albumin level was 3.5 ± 0.6 g/dL. Most patients (70%) had serum AFP levels ≤200 ng/mL. The median number of TACE sessions was two per patient (range: 1–13).

Baseline characteristics were compared between patients who underwent repeated TACE in the prelockdown and lockdown groups (Table 3). The mean repeated TACE interval was significantly longer in the lockdown group than in the prelockdown group (76.7 vs. 63.5 days, P=0.007). However, no significant differences were observed between the two groups in terms of age, sex, liver disease etiology, Child–Pugh class, BCLC staging, levels of serum AFP, ALT, AST, total bilirubin, albumin, and platelet count, size of the main tumor, number of tumors, vascular invasion, previous TACE sessions, or length of hospital stay.

### 3.5. Treatment Response in the Repeated TACE Group

The treatment responses of the two groups are shown in Table 4. The tumor response rates based on the mRECIST criteria in the prelockdown group with complete response (CR), partial response (PR), stable disease (SD), and progressive disease (PD) were 21% (18 patients), 44% (38 patients), 11% (10 patients), and 24% (21 patients), respectively. In the lockdown 2020 group, the CR, PR, SD, and PD response rates were 24% (14 patients), 41% (24 patients), 9% (5 patients), and 26% (15 patients), respectively. The response rates after repeated TACE were not significantly different between the two groups (*P*=0.908). BCLC stage migration was also not significantly different (*P*=0.438). During follow-up, 56 (64.4%) and 34 (58.6%) deaths occurred in the prelockdown and lockdown groups, respectively (*P*=0.600).

### 3.6. Survival of Patients in the Prelockdown and Lockdown Groups

**Figure 2:**
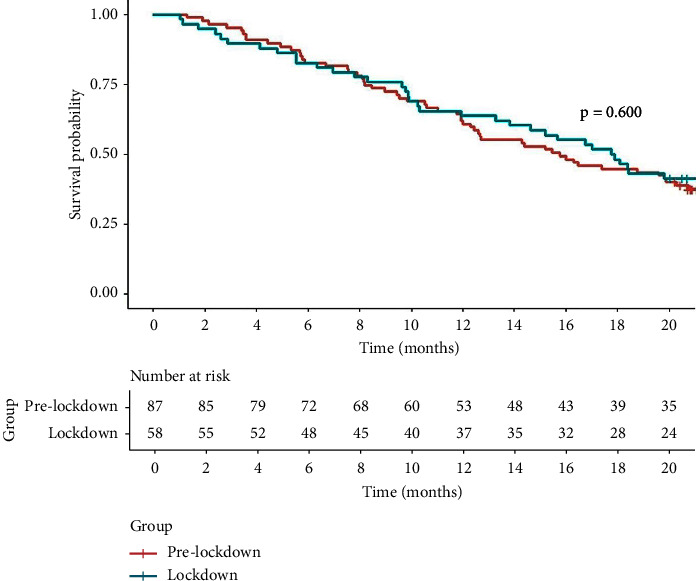
Cumulative survival of HCC patients who underwent repeated transarterial chemoembolization (TACE) during the prelockdown and lockdown periods.

The median OS time of the repeated TACE group was 16.5 months (95% CI 13.8–19.9 months). The median OS times in the prelockdown and lockdown groups were 15.8 months (95% CI 12.4–20.8 months) and 17.8 months (95% CI 13.8‒NA months), respectively. The cumulative survival rates between prelockdown and lockdown groups were not significantly different at 6 months (83% vs. 83%), 12 months (61% vs. 64%), and 18 months (45% vs. 48%) by the log-rank test (*P*=0.600) (Figure 2). The mortality rate was 64.4% (56/87 patients) in the prelockdown group and 58.6% (34/58 patients) in the lockdown group (*P*=0.600).

### 3.7. Prognostic Factors for Survival

Univariate and multivariate logistic regression analyses were used to determine the significant prognostic factors influencing survival (Table 5). Among the 15 factors affecting mortality, univariate analysis revealed that the total bilirubin level >1.0 mg/dL (*P*=0.013), albumin level ≤3.5 g/dL (*P*=0.001), size of main tumor >5 cm (*P*=0.012), number of tumors >5 (*P*  <  0.001), and presence of vascular invasion (*P*  <  0.001) were significant prognostic factors. Multivariate analysis for the potential prognostic factors affecting survival showed that albumin level ≤3.5 g/dL (hazard ratio (HR) 1.73, *P* = 0.016), number of tumors >5 (HR 2.06, *P*=0.002), and presence of vascular invasion (HR 4.59, *P*  <  0.001) were the only three independently significant prognostic factors associated with shorter survival. In univariate and multivariate analyses, the lockdown group and TACE interval >90 days were not significantly associated with decreased survival.

### 3.8. Subgroup of Patients Affected by COVID-19

Of the HCC patients in the repeated TACE group (145 patients) exposed to the pandemic, 2.8% (4/145) (prelockdown (*n* = 2), lockdown (*n* = 2)) had a diagnosis of an active COVID-19 infection. The patients were female in 75% of cases with a mean ± SD age of 58.5 ± 2.2 years. The diagnoses of these COVID-19 patients were based on PCR in three patients (75%). All four patients had only mild symptoms, such as fever, cough, or runny nose, and stayed in home isolation for 10 days. The medical treatment in these patients included an antibiotic regimen and antiviral therapy.

### 3.9. Modifications of Treatment Strategies in the Lockdown Group

During the lockdown period, modifications were made to the TACE treatment during the follow-up period for a total of 17 cases (9 in the repeated TACE group and 8 in the first TACE group). The most frequent reason for modifying the treatment strategy was due to tumor progression, which required a switch to systemic therapy (*n* = 7), best supportive care (*n* = 7), and radiation therapy (*n* = 1). Failed or contraindicated TACE procedures were also a factor, with one patient experiencing unsuccessful tumor feeder catheterization and another patient having severe arterioportal shunting. Notably, no patients required modification to their TACE treatment due to COVID-19-related conditions.

## 4. Discussion

The current study showed a significant decrease in the number of repeated TACE procedures performed among patients diagnosed with inoperable HCC after the COVID-19 outbreak compared to the same period in the previous year. In our study of 262 patients with inoperable HCC, approximately one in seven patients experienced a first TACE delay and nearly one in four experienced a repeated TACE delay. A significant delay of approximately two weeks in repeated TACE procedures was observed in the lockdown group compared to the prelockdown group (76.7 vs. 63.5 days, *P*=0.007). In the multivariate analysis of patients undergoing repeated TACE, the lockdown group and treatment delay >90 days were not associated with increased mortality. Statistically significantly increased mortality hazards were observed in patients with albumin level ≤3.5 g/dL, number of tumors >5, and presence of vascular invasion.

The COVID-19 pandemic has had a significant impact on the delivery of cancer care, and our study highlights some of the challenges faced by HCC patients undergoing repeated TACE therapy. We observed a steep decrease in the number of patients receiving repeated TACE and a delay in the TACE interval time during the pandemic. Furthermore, a significant proportion of patients requiring TACE were not treated at all due to various factors such as postponed procedures, poor liver function, delayed imaging or diagnosis, and limited transportation. The reasons for this delay are multifactorial and include restrictions on hospital visits and triage of patients with COVID-19 infection risks to outdoor quarantine areas. Fear of contracting the virus also led to patient hesitancy in visiting hospitals. Additionally, the COVID-19 pandemic resulted in a shift in the primary attention of healthcare systems towards managing COVID-19 patients, causing routine services in oncology care to receive secondary attention and restricting access to these clinical services. It is important to consider the balance between decreasing the risk of COVID-19 infection and worsening cancer outcomes due to the absence of sufficient cancer treatment, which presents a healthcare ethical dilemma [16, 17]. In settings with limited resources, maintaining routine care for cancer patients during the COVID-19 outbreak should be considered a major priority. These findings highlight the need for strategies to minimize the impact of COVID-19 on cancer care, including telemedicine, home-based care, and appropriate triaging of patients to ensure timely cancer treatment.

We observed that nearly one in four patients experienced a repeated TACE delay. Treatment delays may be anticipated in patients with HCC given the potential changes in liver decompensation and tumor progression [18]. Several studies have shown an association between treatment delays and increased mortality in patients with HCC [6–8]. In our study, patients in the lockdown group or treatment delays >90 days were not associated with survival after adjusting for other prognostic factors. These findings were similar to those of previous studies [9, 10]. There are several reasons underlying these results. First, the selection of patients depends on provider behavior. Patients with HCC who are perceived to have more aggressive tumors are prioritized for treatment but still have a worse prognosis than those with favorable tumor behavior. Second, TACE is a procedure for HCC that is strongly influenced by interventional techniques, especially in selective catheterization. Conventional TACE with a selective technique achieves higher response rates and prolonged survival than lobar TACE [19–21]. Third, patients with HCC in our study had relatively small main tumors and a limited number of tumors. Since nearly 75% of the patients in our study had ≤5 tumor nodules, a favorable outcome was expected in patients who received selective TACE with the possibility of obtaining a good treatment response [22].

At least two sequential TACE procedures should be performed before treatment is abandoned due to a lack of tumor response [23]. In a unilobar disease, repeated TACE sessions will target the same tumor location if imaging findings do not show CR using the mRECIST criteria after the first TACE. In bilobar involvement, each hepatic lobe is targeted for chemoembolization one after another. A panel of experts on the standardization of the lipiodol TACE procedure has recommended performing sequential TACE sessions at 2- to 8-week intervals according to treatment tolerance and efficacy until CR is achieved [24]. If new tumor progression occurs, including treated tumor recurrence or new tumor foci in a nontreated portion of the liver, additional on-demand TACE is indicated [25]. However, the appropriate length of the repeated TACE interval remains controversial. A cross-sectional study on the technical aspects of TACE practice that included 1,160 responses from 62 countries showed that the typical follow-up was between two and four weeks (38%) and four and eight weeks (46%) [26]. Significantly few respondents followed up patients with HCC at less than two weeks or more than two months. Therefore, we propose that a reasonable delay is possible in the TACE interval based on tumor aggressiveness, radiological response, and liver status of patients with HCC.

Low albumin level, number of tumors >5, and presence of vascular invasion were independently associated with shorter survival on multivariate analysis, which was similar to the results of previous studies [22, 27–29]. This agrees with the fact that life expectancy depends not only upon tumor treatment efficacy but also on the underlying severity of liver disease, especially in patients with low albumin levels [27]. Multifocal tumors usually have relatively complex and multiple tumor feeders, making it difficult for TACE to achieve CR [21]. The presence of vascular invasion of portal vein branches occurs in 10–40% of patients with HCC at the initial diagnosis and is associated with a poorer prognosis [28, 29]. Current international guidelines recommend systemic treatment as the first-line option for vascular invasion [12]. Recent data suggested that HCC with vascular invasion might benefit from locoregional therapies alone or a combination of locoregional and systemic treatments [30–32].

The strength of this study is the broad study population with indications for TACE. We included patients with HCC classified with BCLC stages A, B, and C that represented the real-life situation in middle-income countries. Additionally, we evaluated the impact of therapeutic delays in the lockdown group on prognosis consequences not only in radiological response but also on survival outcome. However, our study has some limitations. First, the number of patients in this series was relatively small in a single-center study, and the follow-up duration was relatively short. Second, other technical factors in selective TACE that were not assessed included the grading of portal vein visualization and the safety margin of iodized oil accumulation. Third, our study included only patients in a single institute in southern Thailand, which was mildly impacted by the COVID-19 pandemic. Therefore, the results may not be applicable to patients in areas with a higher prevalence of coronavirus infection.

In conclusion, the COVID-19 pandemic had a significant impact on liver cancer care in southern Thailand, resulting in decreased and delayed delivery of repeated TACE in 2020 compared to 2019. Despite these treatment delays, survival rates for patients with inoperable HCC were not significantly impacted. While these findings do not recommend prolonging waiting times for TACE, a reasonable delay in chemoembolization based on tumor behavior and liver status in patients with HCC does not appear to increase the risk of death.

## Figures and Tables

**Table 1:** Details of TACE interval and TACE delay >90 days between the prelockdown and lockdown groups.

Procedure	Factors	Prelockdown group	Lockdown group	*P* value
First TACE	First TACE interval (days), mean ± SD	46.5 ± 21.6 (*n* = 47)	51.2 ± 28.3 (*n* = 48)	0.826
	First TACE delay > 90 days, *n* (%)	2 (4.3)	7 (14.6)	0.159

Repeated TACE	Repeated TACE interval (days), mean ± SD	63.5 ± 25.2 (*n* = 87)	76.7 ± 32.9 (*n* = 58)	0.007^∗^
	Repeated TACE delay > 90 days, *n* (%)	10 (11.5)	13 (22.4)	0.126

TACE, transarterial chemoembolization; SD, standard deviation.

**Table 2:** Patient and tumor characteristics of the study participants who underwent repeated TACE.

Factors	Value
Patients (*n*)	145
Age, years, mean ± SD	64.0 ± 9.7
Sex, male (%)	109 (75)
Liver disease etiology (%)	
HBV/HCV/HBV + HCV/alcohol/others	55/24/2/6/13
Child–Pugh classification (%)	
A/B	78/22
BCLC stage (%)	
A/B/C	14/73/13
AFP (ng/mL), %	
≤200/>200	70/30
ALT (U/L), median (IQR)	32.0 (23.0–43.0)
AST (U/L), median (IQR)	49.0 (34.0–76.0)
Total bilirubin (mg/dL), median (IQR)	0.8 (0.5–1.3)
Albumin (g/dL), mean ± SD	3.5 ± 0.6
Platelet count (×10^3^/mm^3^), median (IQR)	103.0 (71.0–153.0)
Size of main tumor (cm), median (range)	3.0 (1.1–16.0)
Number of tumors (%)	
1–3/4-5/>5	55/19/26
Presence of vascular invasion, *n* (%)	15 (10)
Previous TACE sessions, median (range)	2 (1–13)
Time interval (days), mean ± SD	68.9 ± 29.3
Hospitalization (days), median (range)	3 (2–40)

TACE, transarterial chemoembolization; SD, standard deviation; HBV, hepatitis B virus; HCV, hepatitis C virus; BCLC, Barcelona Clinic Liver Cancer; AFP, alpha-fetoprotein; ALT, alanine transaminase; IQR, interquartile range; AST, aspartate transaminase.

**Table 3:** Baseline characteristics of the study participants who underwent repeated TACE.

Factors	Prelockdown group (*n* = 87)	Lockdown group (*n* = 58)	*P* value
Age, years, mean ± SD	63.9 ± 10.3	65.3 ± 8.8	0.406
Sex, male, *n* (%)	64 (73.6)	45 (77.6)	
Liver disease etiology, *n* (%)			0.225
HBV	48 (55.2)	31 (53.4)	
HCV	25 (28.7)	10 (17.2)	
HBV + HCV	2 (2.3)	1 (1.7)	
Alcohol	4 (4.6)	5 (8.6)	
Others	8 (9.2)	11 (19)	
Severity of liver disease, *n* (%)			0.079
Child–Pugh A	63 (72.4)	50 (86.2)	
Child–Pugh B	24 (27.6)	8 (13.8)	
BCLC stage, *n* (%)			0.610
A	10 (11.5)	10 (17.2)	
B	65 (74.7)	41 (70.7)	
C	12 (13.8)	7 (12.1)	
AFP (ng/mL), *n* (%)			0.393
≤200	64 (73.6)	38 (65.5)	
>200	23 (26.4)	20 (34.5)	
ALT (U/L), median (IQR)	33.0 (21.5–45.0)	30.5 (25.0–41.0)	0.565
AST (U/L), median (IQR)	55.0 (35.5–76.5)	44.5 (33.0–71.8)	0.218
Total bilirubin (mg/dL), median (IQR)	0.8 (0.5–1.4)	0.9 (0.5–1.3)	0.548
Albumin (g/dL), mean ± SD	3.6 ± 0.6	3.4 ± 0.6	0.165
Platelet count (×10^3^/mm^3^), median (IQR)	112.5 (71.2–149.5)	96 (71.5–153.5)	0.584
Size of main tumor (cm), median (range)	3.2 (1.1–12.8)	3.0 (1.1–16.0)	0.266
Number of tumors, *n* (%)			0.793
1–3	46 (52.9)	34 (58.6)	
4-5	17 (19.5)	10 (17.2)	
>5	24 (27.6)	14 (24.1)	
Vascular invasion, *n* (%)			0.781
Absent	53 (91.4)	77 (88.5)	
Present	5 (8.6)	10 (11.5)	
Previous TACE sessions, median (IQR)	2 (1–5)	2 (1–4)	0.756
Time interval (days), mean ± SD	63.5 ± 25.2	76.7 ± 32.9	0.007^∗^
Hospitalization (days), median (IQR)	3 (3–3.5)	3 (3–3)	0.682

TACE, transarterial chemoembolization; HBV, hepatitis B virus; HCV, hepatitis C virus; BCLC, Barcelona Clinic Liver Cancer; AFP, alpha-fetoprotein; ALT, alanine transaminase; IQR, interquartile range; AST, aspartate transaminase; SD, standard deviation.

**Table 4:** Treatment response analysis at 1 month of prelockdown versus lockdown.

Treatment response	Prelockdown group (*n* = 87)	Lockdown group (*n* = 58)	*P* value
mRECIST at 1 month			0.908
CR	18 (20.7)	14 (24.1)	
PR	38 (43.7)	24 (41.4)	
SD	10 (11.5)	5 (8.6)	
PD	21 (24.1)	15 (25.9)	
BCLC stage migration	3 (3.4)	4 (6.9)	0.438

Data are presented as *n* (%). mRECIST, modified response evaluation criteria in solid tumors; CR, complete response; PR, partial response; SD, stable disease; PD, progressive disease; BCLC, Barcelona Clinic Liver Cancer.

**Table 5:** Univariate and multivariate logistic regression analyses exploring factors associated with survival after chemoembolization.

Prognostic factors	Reference	Univariate analysis		Multivariate analysis	
		HR (95% CI)	*P* value	HR (95% CI)	*P* value
Age > 65 years	≤65 years	0.92 (0.61–1.4)	0.707		
Gender: male	Female	0.80 (0.5–1.27)	0.338		
Etiology					
HCV	HBV	1.34 (0.83–2.17)	0.229		
HBV + HCV		0.40 (0.05–2.87)	0.359		
Others		0.82 (0.45–1.49)	0.518		
Child–Pugh class B	Class A	1.38 (0.86–2.22)	0.182		
AFP level: ≥200 ng/mL	<200 ng/mL	1.26 (0.81–1.96)	0.312		
Total bilirubin: >1.0 mg/dL	≤1.0 mg/dL	1.69 (1.11–2.55)	0.013		
Albumin: ≤3.5 g/dL	>3.5 g/dL	2.04 (1.32–3.16)	0.001	1.73 (1.11–2.71)	0.016^*∗*^
Platelet count: ≤10^5^ mm^3^	>10^5^	1.03 (0.68–1.56)	0.891		
Size of main tumor: >5 cm	≤5 cm	1.90 (1.15–3.14)	0.012		
Number of tumors: >5	≤5	2.31 (1.49–3.57)	<0.001	2.06 (1.32–3.23)	0.002^*∗*^
Vascular invasion: present	Absent	5.08 (2.86–9.02)	<0.001	4.59 (2.55–8.27)	<0.001^*∗*^
Grouping: lockdown	Prelockdown	0.88 (0.57–1.35)	0.555		
Delayed treatment: >90 days	≤90 days	1.25 (0.77–2.02)	0.365		

HR, hazard ratio; CI, confidence interval; HCV, hepatitis C virus; HBV, hepatitis B virus; BCLC, Barcelona Clinic Liver Cancer; AFP, alpha-fetoprotein.

## Data Availability

The datasets used or analyzed during the current study are available from the corresponding author upon reasonable request. In order to protect study participants' privacy, our data cannot be shared openly.
